# Hole-Patterned Pellicles: A Structural Approach for Improved Extreme Ultraviolet Transmittance and Mechanical Behavior

**DOI:** 10.3390/ma19010056

**Published:** 2025-12-23

**Authors:** Haneul Kim, Jungyeon Kim, Young Woo Kang, Taeho Lee, Min-Woo Kim, Tae Joong Ha, Hye-Keun Oh, Jinho Ahn

**Affiliations:** 1 Division of Materials Science and Engineering, Hanyang University, Seoul 04763, Republic of Korea; milky-way@nate.com (H.K.); lbhxlch@naver.com (J.K.); kyw9412@naver.com (Y.W.K.); 2 CH^3^IPS Innovation Research Center, Hanyang University, Seoul 04763, Republic of Korea; taeholee@hanyang.ac.kr; 3 Department of Applied Physics, Hanyang University, Ansan 15588, Republic of Korea; kmw2250@naver.com (M.-W.K.); hyekeun@hanyang.ac.kr (H.-K.O.); 4 TDNJ Inc. TBC SPACE-S, Daejeon 34112, Republic of Korea; tj.ha@tdnj.co.kr

**Keywords:** extreme ultraviolet, pellicle, hole-patterned membrane, porous structure, transmittance, imaging simulation, bulge test

## Abstract

To sustain high-throughput extreme ultraviolet (EUV) lithography, pellicles with high transmittance are essential. As conventional methods—such as material optimization and membrane thinning—have reached their practical limits, alternative strategies are now required. In this study, we investigate an alternative hole-patterned pellicle architecture that introduces a geometric degree of freedom beyond that of continuous-film architectures. EUV transmittance measurements show that transmittance increases with open ratio (OR), following the absorption-limited trend predicted by an OR-based upper bound model, while exhibiting a measurable deviation at higher OR. To provide structural insight into this deviation, pseudo-spectral time domain (PSTD) simulations were performed under scanner-relevant numerical aperture and illumination conditions, solely to extract qualitative angular redistribution trends associated with hole geometry. Lithographic aerial-image simulations indicate that pattern distortion effects emerge only under highly coherent illumination and are suppressed as radius sigma σ_r_ increases. Mechanical characterization using bulge tests reveals distinct pressure–deflection behavior in hole-patterned membranes compared with continuous films, including earlier pressure saturation and modified burst-failure statistics. Although a modest reduction in mean burst pressure is observed, the hole-patterned membranes exhibit a narrower failure distribution, reflecting altered defect sensitivity. Taken together, the results demonstrate how periodic perforation influences transmittance behavior and mechanical response, providing design-relevant trends that complement existing material- and thickness-based pellicle optimization approaches.

## 1. Introduction

Extreme ultraviolet (EUV) lithography is an advanced exposure technology used to fabricate integrated circuits for the 5 nm technology nodes and beyond. Owing to the short wavelength of EUV radiation (13.5 nm), most materials—including air—exhibit strong absorption, necessitating EUV scanner operation under vacuum conditions. However, particles originating from the EUV source and scanner components (e.g., Sn, Cr, Fe) are inevitably generated, may deposit on the reticle, and can be repeatedly imaged onto the wafer, leading to yield loss [1]. Consequently, effective protection of the reticle from particles is essential for high-yield EUV lithography [2].

To mitigate this contamination problem, an EUV pellicle—a nanometer-scale free-standing membrane—can be employed to hold fall-on particles out of focus while transmitting EUV light. Nonetheless, any residual absorption in the membrane reduces the effective dose, impacting the scanner throughput; hence, pellicle transmittance targets exceeding 90% have been widely pursued [3]. With conventional improvement technologies, such as material optimization at 13.5 nm and further membrane thinning, reaching their practical limits, the development of alternative strategies has become crucial [4,5].

One structural pathway is to re-engineer the free-standing membrane itself. For example, prior studies have explored surface nanostructuring (e.g., DSA-patterned SiN_x_ with partial surface etching), but the relatively thick membranes (~110 nm) and non-through features limited transmittance (~60%) [4]. Grid-supported, micrometer-thick honeycomb models improved robustness but introduced transmittance non-uniformity and exhibited negligible thermal/mechanical benefits in simulations [6].

Recent progress in EUV pellicle development has increasingly focused on metal-silicide materials such as MoSi_2_ and ZrSi_2_, owing to their favorable EUV optical constants and high thermal emissivity [7,8]. Despite these material advances, all state-of-the-art pellicles retain a continuous-film architecture and therefore remain constrained by thickness-dependent absorption and mechanical behavior governed by a continuous-film stress distribution. Prior studies have not explored whether introducing structural perforation—beyond material or thickness optimization—could serve as an additional design degree of freedom influencing the transmittance and mechanical behavior.

These considerations motivate the exploration of structurally perforated membranes as a complementary design strategy beyond conventional material- and thickness-based optimization.

In this study, we designed and fabricated periodically arranged through-hole arrays in a Si-rich SiN_x_ pellicle and experimentally examined how the open ratio (OR) influences the transmittance relative to the absorption-limited trend predicted by an OR-based upper bound model. Independently of this experimental comparison, pseudo-spectral time domain (PSTD) simulations were performed under scanner-relevant numerical aperture (NA) and illumination conditions to extract qualitative angular redistribution trends associated with hole geometry in relation to the OR-based upper bound model. Mechanical characterization using bulge tests revealed a distinctive early differential-pressure saturation in perforated membranes, offering insight into their vacuum-loading behavior in comparison with continuous films. By integrating experimental measurements with simulation-based structural analysis, this work provides design-relevant trends on how geometric parameters of periodic perforations influence effective transmittance, complementing material- and thickness-based optimization pathways.

## 2. Materials and Methods

### 2.1. Geometric Design and Fabrication of Hole-Patterned EUV Pellicles

EUV pellicles must meet stringent requirements, including high transmittance, low transmittance non-uniformity, low reflectance, and high thermo-mechanical stability. Among these, a high transmittance is particularly important to minimize the throughput loss during the exposure process [9]. To evaluate the influence of structural parameters on transmittance, periodic hole arrays with different arrangements—triangular, square, and honeycomb—were investigated, each defined by the hole diameter (*d*) and spacing (*s*). For quantitative comparison among the array types, a unit surface—defined as the smallest repeating element that could reconstruct the entire array through symmetry—was adopted. The OR was defined as the ratio of the perforated area to unit surface area [10]. Among the three configurations, the triangular array exhibited the largest OR under identical dimensional conditions. Consequently, it was expected to provide the greatest potential for enhancing transmittance. Accordingly, triangular arrays were selected for experimental validation.

Accordingly, 37 nm thick SiN_x_ films were deposited via low-pressure chemical vapor deposition (LPCVD) at 810 °C using dichlorosilane (SiH_2_Cl_2_) and ammonia (NH_3_) as reactive gases. Next, a 10 × 10 mm^2^ hole-pattern region was formed by ArF dry lithography on an ASML XT1250 scanner, followed by reactive ion etching (RIE) [11]. Subsequently, a SiO_2_ layer was deposited to protect the patterned region.

**Figure 1:**
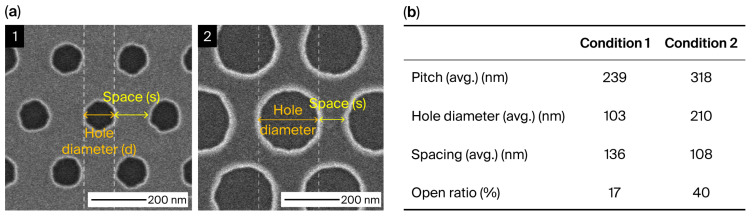
(**a**) Top-down CD-SEM images of the triangle array after hole etching. (**b**) A table summarizing the key parameters and the OR based on measured hole diameter and spacing.

Figure 1a presents critical-dimension scanning electron microscopy (CD-SEM) images obtained after hole etching. The measured hole diameter and spacing were used to calculate the experimental ORs for each design condition (Figure 1b). Specifically, Condition 1 exhibited a hole diameter of 103 nm with a spacing of 136 nm (OR = 17%), whereas Condition 2 exhibited a hole diameter of 210 nm with a spacing of 108 nm (OR = 40%).

Cross-sectional transmission electron microscopy and surface atomic force microscopy analyses confirmed an LPCVD SiN_x_ membrane thickness of approximately 37 nm and a surface roughness with an RMS value of ~0.3 nm (Supplementary Figure S1). The film thickness uniformity across the wafer area was within 1.5%.

To fabricate free-standing 10 × 10 mm^2^ membranes, exposure, development, and RIE were sequentially applied to the backside of the wafer. The underlying silicon substrate and SiO_2_ layer were then removed simultaneously by wet etching in a 30 wt% potassium hydroxide (KOH) solution at 60 °C, with a water bath used to maintain stable temperature conditions throughout the etching process [12]. The final free-standing SiN_x_ membrane had a thickness of 37 nm. A temporary SiO_2_ protection layer was used to prevent KOH-induced SiN_x_ thinning and to suppress hydrogen-bubble-related damage generated when the exposed Si of the front-side hole-pattern region is etched during the wet etch process. The released membrane was supported by the surrounding Si frame.

### 2.2. Optical Characterization and Analytical–Numerical Modeling of Hole-Patterned Pellicles

The optical characteristics of the hole-patterned pellicles were examined using synchrotron-based transmission measurements, an OR-based analytical calculation, and numerical simulations. The incident angle for all EUV measurements was fixed at 6°, consistent with the illumination geometry of EUV scanners. Transmittance measurements were performed at the Pohang Accelerator Laboratory, Beamline 4B1 (Pohang, Republic of Korea), using monochromatic radiation centered at 13.5 nm with a spectral bandwidth of 0.1%. The EUV beam diameter at the sample plane was 400 µm. Although this spot size does not illuminate the entire 10 × 10 mm^2^ area simultaneously, the pellicle was mounted on a motorized translation stage, enabling positioning so that different regions of the pellicle could be probed as required. For the analytical comparison, the effective transmittance was estimated using an OR-based model that provides an absorption-limited geometric upper bound. The experimentally measured transmittance values were compared with this upper bound to examine how perforation-induced geometric transparency scales with OR.

To examine the qualitative angular redistribution behavior associated with the perforated geometry, PSTD simulations were performed in Fastlitho [13]. The optical constants (n and k) of SiN_x_ used in the simulations were obtained from the Center for X-Ray Optics database. The simulation model incorporated film thickness of 18 nm and material optical constants, along with a wavelength of 13.5 nm, an incidence angle of 6°, and a pellicle standoff distance of 2.5 mm, to represent in-scanner conditions. Two simulation sequences were implemented to evaluate the angular redistribution and the impact on aerial-image distortion.

In the first simulation sequence, a uniform conventional illumination source with σ_r_ = 0.2 (expressed here as radius sigma σ_r_ for consistency with later dipole cases) and a 0.33 NA projection system—representative of typical EUV scanner optics—was used to compute the angular distribution of EUV light propagating through the hole-patterned membrane. The simulated angular distribution was evaluated relative to the absorption-limited OR model, which provides the geometric upper bound in the absence of angular redistribution. The deviation from this upper bound was used to extract the qualitative trend of NA-out angular redistribution associated with changes in hole geometry [14].

In the second simulation sequence, lithographic aerial-image simulations were performed using the same 0.33 NA projection optics. For the 24 nm half-pitch contact-hole (C/H) pattern, a uniform conventional illumination pupil was used, and for the 12 nm half-pitch line-and-space (L/S) pattern, a uniform dipole illumination pupil with center sigma (σ_c_) = 0.6 was applied.

To examine how illumination coherence affects imaging performance, the illumination pupil σ_r_ was systematically varied from 0 to 0.0006 for both pattern types.

This sweep isolates the influence of highly coherent illumination conditions on aerial-image contrast and pattern fidelity in the presence of the hole-patterned membrane. The simulation parameters and material constants used in this study are summarized in Table 1.

### 2.3. Mechanical Characterization of Free-Standing Membranes via the Bulge Test

**Figure 2:**
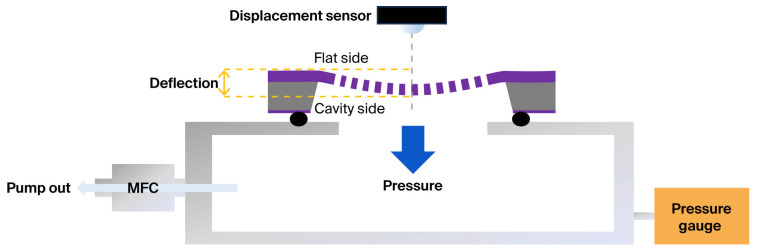
Schematic of the bulge test equipment.

Figure 2 shows a schematic of the bulge-test setup used to evaluate the mechanical properties of the free-standing membrane [15,16]. In this setup, the chamber pressure was controlled by adjusting the pumping flow rate through a mass flow controller, thereby generating a uniform pressure differential across the membrane. During testing, the specimen was mounted with its cavity side facing the pressure source, such that the applied differential pressure induced deflection toward the cavity side rather than the flat side. In this study, the entire 10 × 10 mm^2^ free-standing membrane was subjected to mechanical loading, as the uniform pressure field generated by the bulge test acts over the full membrane area. The term ‘cavity side’ denotes the concave surface of the Si frame formed during backside etching and is used to indicate the orientation in which the membrane is mounted in the bulge-test fixture. In this configuration, the applied pressure naturally acts toward the cavity side due to the layout of the test chamber and membrane holder. This configuration was adopted to mitigate the singular stress concentration that develops at the sharp corner between the supporting frame and the membrane, which can otherwise cause interfacial delamination or premature failure [17]. By minimizing the effect of interfacial adhesion between the membrane and the supporting frame, the reliability of the fracture-strength measurements could be improved.

To analyze the deflection and pressure-relief behavior of the membrane, the pumping speed was systematically varied to generate controlled pressure gradients across the film. The central deflection of the membrane was continuously monitored in real time using a laser displacement sensor (OMRON Corporation, Kyoto, Japan).

## 3. Results and Discussion

### 3.1. Transmittance Characteristics and Comparison with an Analytical Upper Bound Model

The transmittance ($T_{p}$) of an unpatterned SiN_x_ pellicle can be calculated under the oblique incidence of EUV light using the Beer–Lambert law, as follows:

(1) $$T_{p}=e^{-\alpha d}$$

where *α* = 4π*k*/*λ* is the absorption coefficient defined by the extinction coefficient *k*, the film thickness *d*, and the EUV wavelength *λ*.

For perforated membranes, the geometric OR provides an upper bound estimate of the fraction of light that bypasses the absorbing material. Under this assumption, the effective transmittance $T_{e}$ of the hole-patterned pellicle can be written as

(2) $$T_{e}=T_{p}+\left[1-T_{p}\right]\cdot open\;ratio\left(OR\right),$$

which represents a purely geometric absorption-limited upper bound that neglects all non-absorptive loss channels.

**Figure 3:**
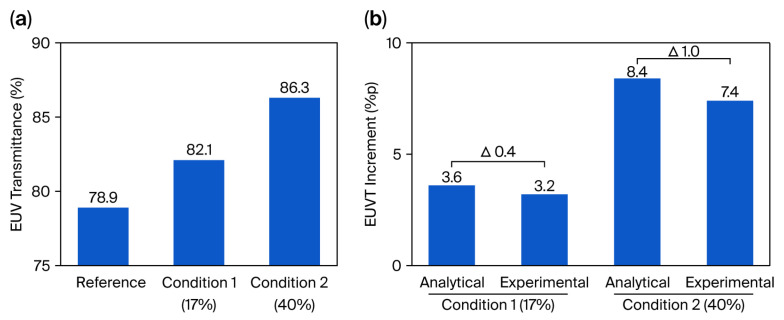
(**a**) EUV transmittance measurement results for the hole-patterned pellicle. Under Condition 1 (OR of 17%), a 3.2 percentage point increase was evident, whereas Condition 2 (OR of 40%) resulted in a 7.4 percentage point increase. (**b**) Comparison of the EUV transmittance increment between the analytical OR-based upper bound model and experimental measurements of Conditions 1 and 2. The symbol △ denotes the difference between the analytical OR-based model and the experimentally measured transmittance increment.

Figure 3a shows the measured transmittance for the 37 nm thick SiN_x_ reference pellicle (78.9%), as well as for hole-patterned pellicles with OR = 17% (Condition 1) and OR = 40% (Condition 2), which achieved 82.1% and 86.3%, respectively. Introducing perforations therefore yields increases of 3.2 and 7.4 percentage points at the same thickness. These results reveal that the transmittance scales with OR, confirming that periodic perforation enables a structural degree of freedom for improving throughput that is not accessible in continuous-film architectures without reducing thickness. Although the achieved 86.3% transmittance is still below the ≥90% target of current HVM pellicles, it is obtained without thickness reduction, and thus represents a structural gain that can be combined with ongoing material and thickness optimization.

Figure 3b compares the measured transmittance values with those predicted by the analytical OR-based absorption-limited upper bound model. Under both conditions, the measured transmittance lies below this geometric upper limit, indicating the presence of additional non-absorptive mechanisms in real membranes. This experimentally observed deviation arises from a combination of effects—including 3D structural features of the perforations, non-ideal hole geometry or surface roughness variations, high-angle diffraction or forward-scattering components, and minor measurement-related uncertainties—which collectively reduce the measured throughput relative to the idealized bound. Importantly, this deviation is not interpreted as an isolated or quantitatively calibrated “scattering factor,” but rather as a qualitative indicator that perforation-induced non-absorptive losses increase modestly with OR.

To further investigate how geometric parameters influence the redistribution of EUV light into angles beyond the NA of the projection optics, PSTD simulations were performed under scanner-relevant illumination conditions. Although not intended to quantitatively reconstruct the experimental deviation, the simulations were carried out using hole diameters, pitches, and OR values constrained by practical pellicle requirements [3]. This ensured that the simulated geometries remained consistent with particle-blocking criteria while enabling extraction of qualitative NA-out redistribution trends. These insights complement the experimental data and provide design guidance for experimentally relevant perforated-pellicle configurations.

### 3.2. Qualitative Angular Redistribution Analysis Using PSTD Simulations

To assess how perforation influences EUV propagation under scanner-relevant illumination conditions, simulations were performed independently of the synchrotron measurements. The EUV scanner imposes a finite NA, such that any angularly redistributed component lying outside the collection cone manifests as an effective loss. Accordingly, these simulations were conducted to extract qualitative angular redistribution trends that inform pellicle design, rather than to reconstruct the experimentally observed deviation from the OR-based upper bound model.

Because EUV projection optics operate at 4× reduction, mask-side defects smaller than ~50 nm fall below the printability threshold for contemporary EUV technology [3]. For this reason, simulated hole diameters were restricted to ≤50 nm, ensuring that the modeled perforations remained non-printing while allowing for controlled evaluation of angular redistribution behavior. Two simulation sets were designed to decouple the effects of the OR and the hole diameter. In the first set, the hole diameter was fixed at 50 nm while the OR was varied to evaluate the effect of array density. In the second set, the OR was maintained at 17%, and the hole diameter was systematically varied to isolate size-dependent redistribution behavior [14].

**Figure 4:**
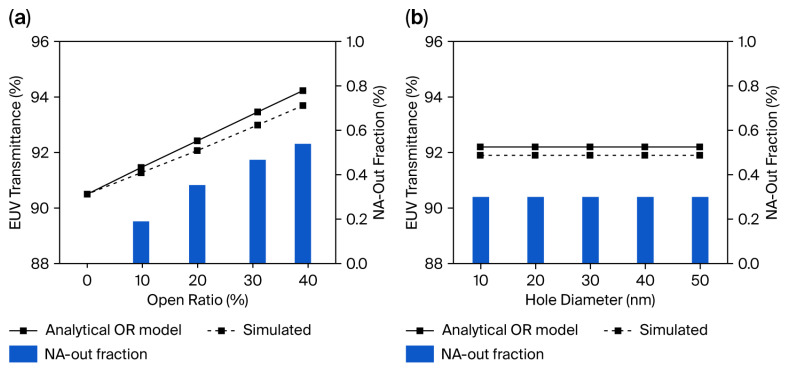
Comparison of the analytical OR-based upper bound model and simulated EUV transmittance, together with the corresponding NA-out fraction, plotted as a function of the (**a**) OR (hole diameter = 50 nm) and (**b**) hole diameter (OR = 17%).

Figure 4 presents the results of the analytical OR-based calculations and numerical PSTD-based simulations. In this analysis, the difference between the transmittance predicted by the OR-based analytical model (Equation (2)) and the value obtained from optical simulations is used as a qualitative indicator of the fraction of EUV light redistributed outside the 0.33 NA of the projection optics, rather than as a quantitatively calibrated scattering metric.

As shown in Figure 4a, where the hole diameter is fixed at 50 nm, increasing OR results in a gradual rise in the fraction of light redistributed into angles lying outside the 0.33 NA collection cone. This trend arises because higher OR values correspond to narrower web spacing and reduced array periodicity, which enhances wide-angle diffraction components. The increased density of the scattering centers thus promotes stronger angular redistribution, even though the absolute magnitude remains modest within the practical OR range considered for pellicle design.

In Figure 4b, where OR is fixed at 17%, varying the hole diameter produces only minor changes in the redistributed fraction. This weak dependence can be qualitatively understood as a compensating interplay between single-aperture and array-level effects [18]. While increasing the hole diameter modifies the single-aperture diffraction characteristics, maintaining a constant open ratio necessarily reduces the spatial density of holes, thereby diminishing the collective contribution of the array to wide-angle redistribution [19]. As a result, these opposing trends partially offset each other, leading to a redistribution level that remains nearly constant over the investigated diameter range. This behavior indicates that, within realistic pellicle-design constraints, the open ratio serves as the structural parameter governing angular redistribution, whereas moderate variations in sub-50 nm hole diameter exert a secondary influence.

It is important to emphasize that the angular redistribution fraction plotted in Figure 4 does not represent a quantitatively deconvolved “scattering loss,” nor is it used to reconstruct the deviation observed experimentally in Section 3.1. Instead, it serves as a qualitative indicator of how perforation geometry influences NA-limited transmittance within an EUV scanner. These simulation trends complement the experimental results by showing that modest redistribution effects arise primarily from increased OR, whereas variations in hole diameter (within the non-printing regime ≤ 50 nm) have a comparatively minor impact.

### 3.3. Impact of Hole-Patterned Pellicles on Imaging Distortion

For the imaging analysis, the perforated membrane geometry was set to hole diameter = 50 nm, OR = 17%, consistent with the non-printing regime discussed in Section 3.2. To evaluate the imaging feasibility of the hole-patterned pellicles, PSTD simulations were extended to generate aerial images under scanner-representative conditions. All simulations were performed using a 0.33 NA, 4×-reduction EUV projection system with a 2.5 mm pellicle–mask standoff distance, consistent with the conditions defined in Section 2.2. Conventional illumination was used for the 24 nm half-pitch (HP) contact/hole (C/H) pattern, whereas a circular dipole source with center sigma σ_c_ = 0.6 was applied for the 12 nm HP line-and-space (L/S) pattern. To probe the sensitivity of aerial images to illumination coherence, the pupil radius sigma (σ_r_) was first swept over an artificially low range (σ_r_ = 0–0.0006) within the PSTD framework. Here, perfectly coherent illumination (σ_r_ = 0) was employed solely to test whether any pellicle-induced shadowing or interference artifacts could emerge under an extreme worst-case condition, rather than to represent a realistic EUV scanner illumination setting.

**Figure 5:**
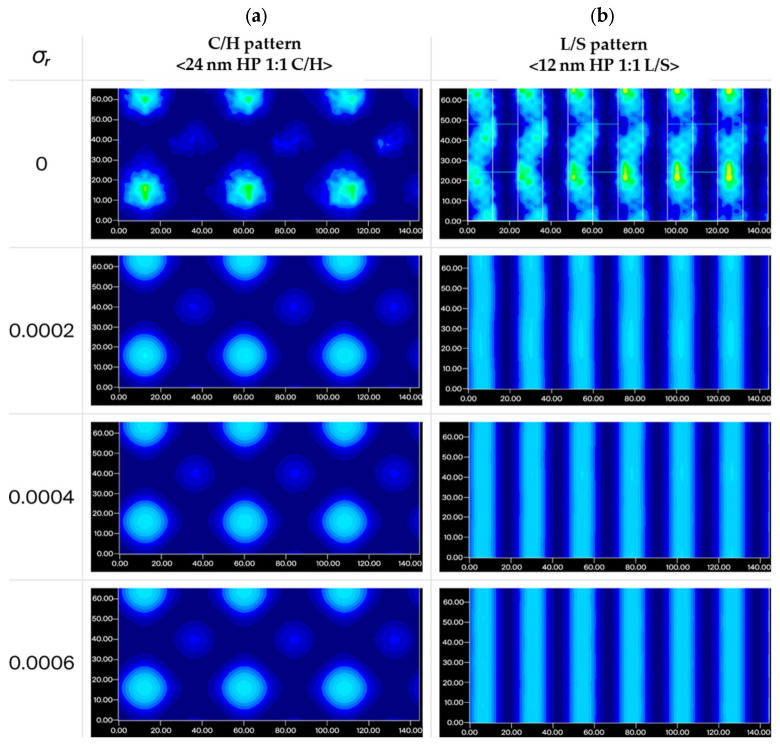
Simulated aerial images of the (**a**) 24 nm half-pitch contact/hole (C/H) pattern and (**b**) 12 nm half-pitch line-and-space (L/S) pattern depending on the radius sigma (*σ_r_*).

As shown in Figure 5, under fully coherent illumination (σ_r_ = 0), both the C/H and L/S patterns exhibit noticeable distortion, including interference fringes, localized intensity modulations, and blurring of feature boundaries. These coherence-driven artifacts originate from the periodic circular-hole geometry of the pellicle, which supports azimuthally symmetric diffraction components. Under highly coherent illumination, the resulting interference field adopts a rotationally symmetric form, producing ring-like intensity modulations that are largely independent of the underlying mask pattern. As σ_r_ is increased within the sensitivity sweep, the visibility of these interference-related features decreases markedly, indicating that relaxing coherence reduces structure-induced interference effects in the simulated images.

It is important to emphasize that the σ_r_ values explored in this sweep serve solely as a sensitivity analysis within the PSTD framework and do not correspond directly to practical scanner pupil settings. Modern EUV scanners operate with substantially larger effective pupil radii, depending on the illumination mode, under which coherence-driven interference effects would likely be further averaged out.

Taken together, these results indicate that, within the simulated cases examined, the coherence-driven artifacts identified under the extreme coherent limit are not sustained as illumination coherence is relaxed, and no obvious additional spatial distortion attributable to the hole-patterned pellicle was observed in the PSTD-based imaging analysis.

To further check consistency with representative scanner illumination conditions, additional aerial-image simulations were performed for selected pupil radius sigma values (σ_r_ = 0.2, 0.4, 0.6, and 0.8 for conventional, σ_r_ = 0.1, 0.2, 0.3, and 0.4 for dipole), with and without the hole-patterned pellicle. As shown in Supplementary Figure S2, no obvious additional distortion attributable to the hole-patterned pellicle was observed in the simulated aerial images across the examined σ_r_ range. This observation is consistent with the trend in Figure 5, where coherence-driven interference features identified under the extreme coherent limit diminish as illumination coherence is relaxed.

### 3.4. Evaluation of Mechanical Characteristics via the Bulge Test

**Figure 6:**
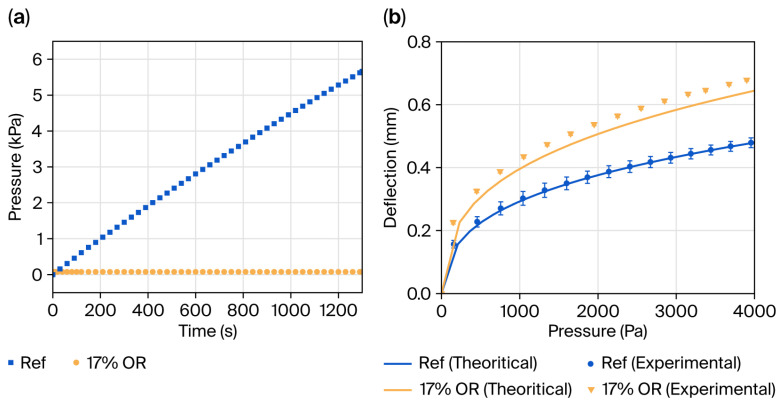
Bulge-test measurements: (**a**) The fixed-flow pump-down condition. (**b**) The pressure–deflection curve under an equivalent pressure-increase rate of reference and 17% OR pellicle. The calculated value Via the square-membrane bulge model is overlaid for comparison.

Bulge tests were performed to compare the pressure–deflection behavior of an unpatterned SiN_x_ (reference) pellicle and a hole-patterned pellicle with a 17% OR (Condition 1). Figure 6a shows the measured deflection under a constant pressure ramp rate of 4.3 Pa·s^−1^, generated while maintaining a 100 sccm flow rate. The reference pellicle exhibited a linear pressure increase followed by rupture at a differential pressure of approximately 5800 Pa. By contrast, the hole-patterned pellicle displayed an early saturation of the pressure difference at approximately 78 Pa, maintaining structural stability without rupture.

Importantly, this self-limiting pressure response highlights a mechanical aspect relevant to one of the challenges in EUV pellicle operation, namely failure during vacuum initialization. Whereas a continuous-film membrane can experience abrupt stress accumulation owing to sealed pressure differentials, a perforated configuration allows partial pressure equilibration under the applied evacuation conditions. As a result, perforated membranes exhibit a distinct pump-down pressure–deflection behavior, not observed in continuous-film pellicles, that reduces the ΔP buildup and can suppress the propensity for ΔP-driven fracture behavior during vacuum loading. These findings indicate that introducing an open structure offers a geometric benefit for tailoring the differential-pressure response in pellicle design [17,20,21].

Figure 6b presents the pressure–deflection curves obtained by controlling the differential pressure at a constant rate for two distinct conditions. The measured deflections were subsequently compared against the square-membrane bulge model. The model employed for this analysis is based on the work of Vlassak and Nix, and the corresponding governing equation can be expressed as follows [15]:

(3) $$q=C_{1}\sigma t\frac{h}{a^{2}}+C_{2}Mt\frac{h^{3}}{a^{4}}$$

where *q* denotes the pressure difference; *a* denotes the half length of the membrane edge; *M* denotes the biaxial modulus defined as *M* = *E*/(1 − *ν*), with *E* (Young’s modulus); *t* denotes the membrane thickness; σ denotes residual stress; and *h* denotes the vertical displacement at the center of the membrane. The coefficients *C*_1_ and *C*_2_ depend on the membrane geometry and Poisson’s ratio and were taken to be 3.393 and (0.792 + 0.0085ν)^−3^, respectively [15].

For model fitting, typical parameters for LPCVD Si-rich SiN_x_ were used (*E* = 200 GPa, ν = 0.25), resulting in a fitted residual stress of 100 MPa for the unpatterned membrane with a coefficient of determination R^2^ > 0.99, consistent with our previous research [22,23].

Using the same validated material parameters, the hole-patterned pellicle was fitted by applying effective elastic properties (*E_eff_*, *σ_eff_*) to account for the porosity [24,25,26]. However, even with these adjustments, the model under-predicted deflection by ~10% across the measured range, indicating higher compliance than predicted.

This discrepancy originates from the limitations of the classical bulge model, which assumes a homogeneous, impermeable film. Perforation reduces the load-bearing area and redistributes stress into a web-like network, effectively lowering the membrane stiffness. In homogenization terms, the elastic modulus becomes *E_eff_* < *E*, reducing the cubic stiffness term and increasing deflection [26].

Additionally, the circular holes introduce free edges that generate tangential stress concentration at the rims, locally amplifying the strain and effectively increasing local compliance, which contributes to enhanced deformation under biaxial inflation [27,28,29]. Consequently, directly applying dense-film coefficients *C*_1_ and *C*_2_ to perforated membranes can lead to overestimation of the rigidity.

**Figure 7:**
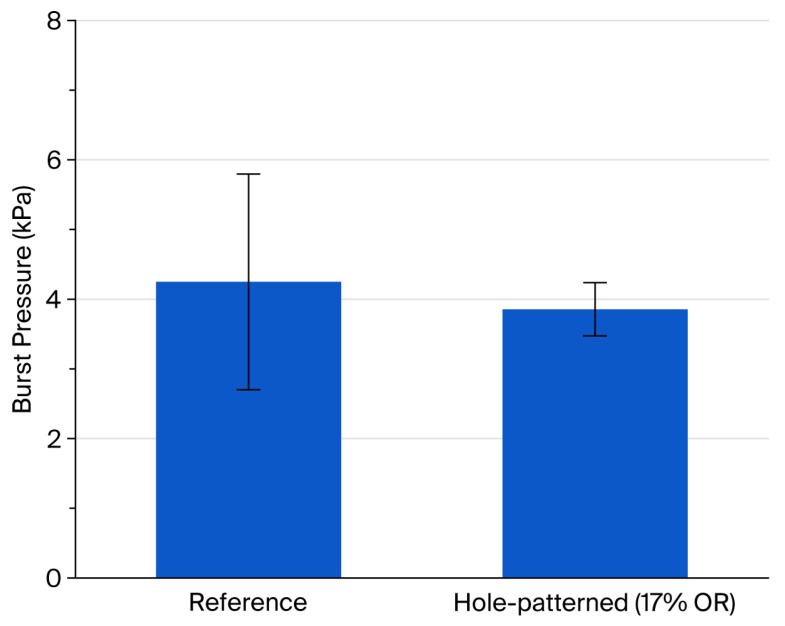
Burst pressure comparison of reference and 17% OR hole-patterned SiN_x_ pellicles.

Figure 7 shows the burst pressure of the same samples whose test results are displayed in Figure 6. The reference exhibited an average burst pressure of 4249 Pa, whereas the hole-patterned specimen failed at 3856 Pa (i.e., exhibiting an approximately 9% lower mean strength). This reduction was expected because perforation introduced free edges at each hole, producing local stress concentration and a smaller net load-bearing cross-section. Consequently, at a given differential pressure, the web regions experienced higher peak stress, promoting earlier fracture [29].

Notably, the standard deviation was larger for the reference than for the hole-patterned pellicle. This could be attributed to the weakest-link sensitivity of a 10 × 10 mm^2^ dense membrane: incidental defects or pinholes act as uncontrolled stress concentrators, leading to premature, highly variable failure events [30]. By contrast, the hole-patterned membrane already contains a uniform, periodic array of perforations that distributes load through repeated web elements; as a result, fracture tends to initiate at geometrically predictable locations and the response becomes less sensitive to random, isolated defects, yielding a smaller dispersion in burst pressure.

From a statistical standpoint, this distinction highlights differences in failure behavior between perforated and continuous membranes at the coupon scale. While the hole-patterned membranes exhibit a modest reduction in mean burst pressure owing to intrinsic stress concentration at hole edges, the narrower failure distribution indicates reduced sensitivity to stochastic defects under the tested conditions. This trade-off—lower mean strength accompanied by reduced variability—clarifies how perforation geometry influences burst-failure statistics in perforated membranes.

Nonetheless, we emphasize that these conclusions are drawn from mechanical measurements performed solely on 10 × 10 mm^2^ coupon-scale membranes. The realization, fabrication, and examination of a full 110 × 144 mm^2^ hole-patterned pellicle will require additional process scaling, stress-management optimization, and dedicated reliability assessment.

## 4. Conclusions

This paper presents a hole-patterned EUV pellicle architecture that introduces a degree of freedom in the geometric design beyond that of conventional continuous-film membranes. Synchrotron-based measurements showed that transmittance increases with the open ratio (OR) without modifying material composition or film thickness. These results follow the absorption-limited trend predicted by an OR-based upper bound model, and the experimentally observed deviation from this idealized limit remained modest across the tested perforation levels.

To complement the measurements, PSTD simulations were performed independently to examine qualitative angular redistribution behavior under scanner-relevant numerical aperture and illumination conditions. These simulations were not used to quantitatively reconstruct the measured deviation, but to identify structural trends—such as increasing redistributions with larger ORs—that provide design-relevant insights into how perforation geometry influences in-scanner EUV propagation.

Mechanical characterization revealed that perforated membranes exhibited an early differential-pressure saturation during pump-down, whereas continuous films experienced continued pressure buildup, leading to failure. This behavior suggests a distinct vacuum-loading response associated with the perforated architecture. Although burst tests showed a modest reduction in mean strength, the failure distribution narrowed, indicating reduced sensitivity to stochastic defects compared with continuous-film membranes.

Finally, it should be emphasized that the present study does not aim to qualify a specific pellicle material system for direct EUV scanner deployment. Metrics such as low reflectance (≤0.04%), thermal stability under ≥400 W source power, and long-term chemical resistance depend on material chemistry and process integration and are therefore beyond the scope of this geometry-focused investigation. While perforation may, in principle, reduce the effective reflective area of a membrane, quantitative assessment of such effects requires measurement accuracy beyond the present experimental capability. These aspects are identified as important directions for future work, in which the perforation geometries established here can be combined with low EUV reflectance, thermally robust material systems and advanced materials optimization strategies.

Overall, this work establishes an integrated experimental–analytical framework that links OR engineering, qualitative scanner-based optical assessment, and comparative mechanical evaluation. The findings demonstrate that structural perforation expands the geometric design space of EUV pellicles beyond material optimization and thickness reduction alone and provide design-relevant trends that may inform future studies of hole-patterned pellicle architectures.

## Figures and Tables

**Table 1:** Simulation parameters and material constants.

Parameter	Value
Pattern half-pitch	24 nm (C/H) 12 nm (L/S)
Illumination source shape	Conventional (C/H) Dipole (L/S)
EUV pellicle material	SiN_x_
Refractive index of the pellicle (n)	0.9832
Extinction coefficient of the pellicle (k)	0.0059
EUV pellicle thickness	18 nm
Magnification	×4
Standoff distance	2.5 mm
Numerical aperture (NA)	0.33

## Data Availability

The data presented in this study are available on request from the corresponding author upon reasonable request. The data are not publicly available owing to privacy issues and conflicts of interest.

## Supplementary Materials

The following supporting information can be downloaded at: https://www.mdpi.com/article/10.3390/ma19010056/s1, Figure S1: (a) Cross-sectional transmission electron microscopy image of the LPCVD SiN_x_ film, showing a uniform thickness of approximately 37 nm. (b) Atomic force microscopy surface topography of the SiN_x_ film, exhibiting a low surface roughness with a root-mean-square value of 0.3 nm; Figure S2: Simulated aerial images for representative scanner-relevant pupil radius sigma values (σ_r_ = 0.2, 0.4, 0.6, and 0.8 for contact/hole (C/H), σ_r_ = 0.1, 0.2, 0.3, and 0.4 for line-and-space (L/S)), comparing cases with and without the hole-patterned pellicle for (a) 24 nm half-pitch C/H and (b) 12 nm half-pitch line-and-space (L/S) patterns. The different σ_r_ ranges reflect the distinct dipole illumination conditions used for the two pattern types, arising from differences in the selected dipole center-sigma (σ_c_ = 0.6) settings. While the introduction of hole-patterned pellicle results in a reduced overall aerial-image intensity due to finite transmittance, no distinct distortion associated with coherent-interference effects—such as those observed under near-zero sigma conditions in Figure 5—was detected across the investigated σ_r_ range.

## Abbreviations

The following abbreviations are used in this manuscript:

CD-SEM	Critical-dimension scanning electron microscopy
EUV	Extreme ultraviolet
LPCVD	Low-pressure chemical vapor deposition
NA	Numerical aperture
OR	Open ratio
PSTD	Pseudo-spectral time domain
RIE	Reactive ion etching
