# Supplementary material for: Hole-Patterned Pellicles: A Structural Approach for Improved Extreme Ultraviolet Transmittance and Mechanical Behavior
Source: Materials (Basel). 2025 Dec 23;19(1):56. doi: 10.3390/ma19010056 (PMC12786459; doi:10.3390/ma19010056)
Supplement: Supplementary file 1 [file materials-19-00056-s001.zip › materials-3993068-supplementary.pdf]

Supporting Information for

Hole-Patterned Pellicles: A Structural Approach for Improved Extreme Ultraviolet Transmittance and Mechanical Behavior

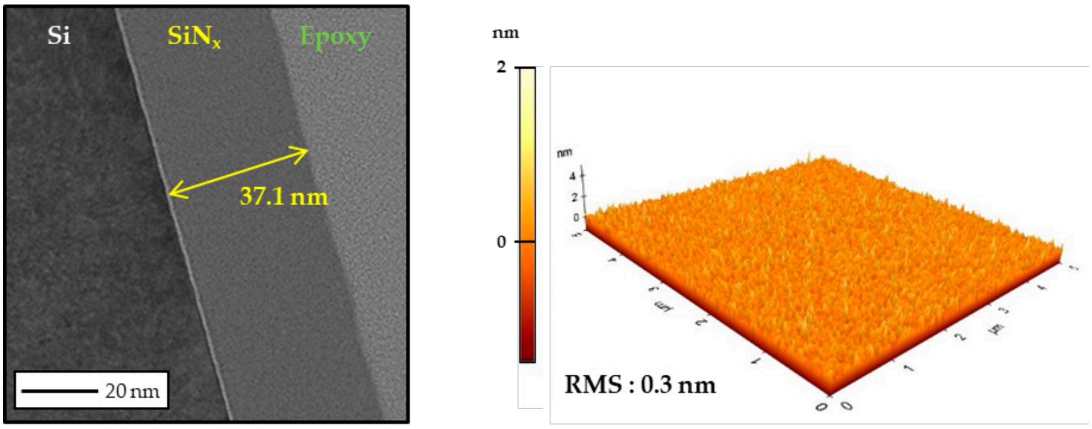

(a) **(b)**

Figure S1. (a) Cross-sectional transmission electron microscopy image of the LPCVD SiN_x film, showing a uniform thickness of approximately 37 nm. (b) Atomic force microscopy surface topography of the SiN_x film, exhibiting a low surface roughness with a root-mean-square value of 0.3 nm.

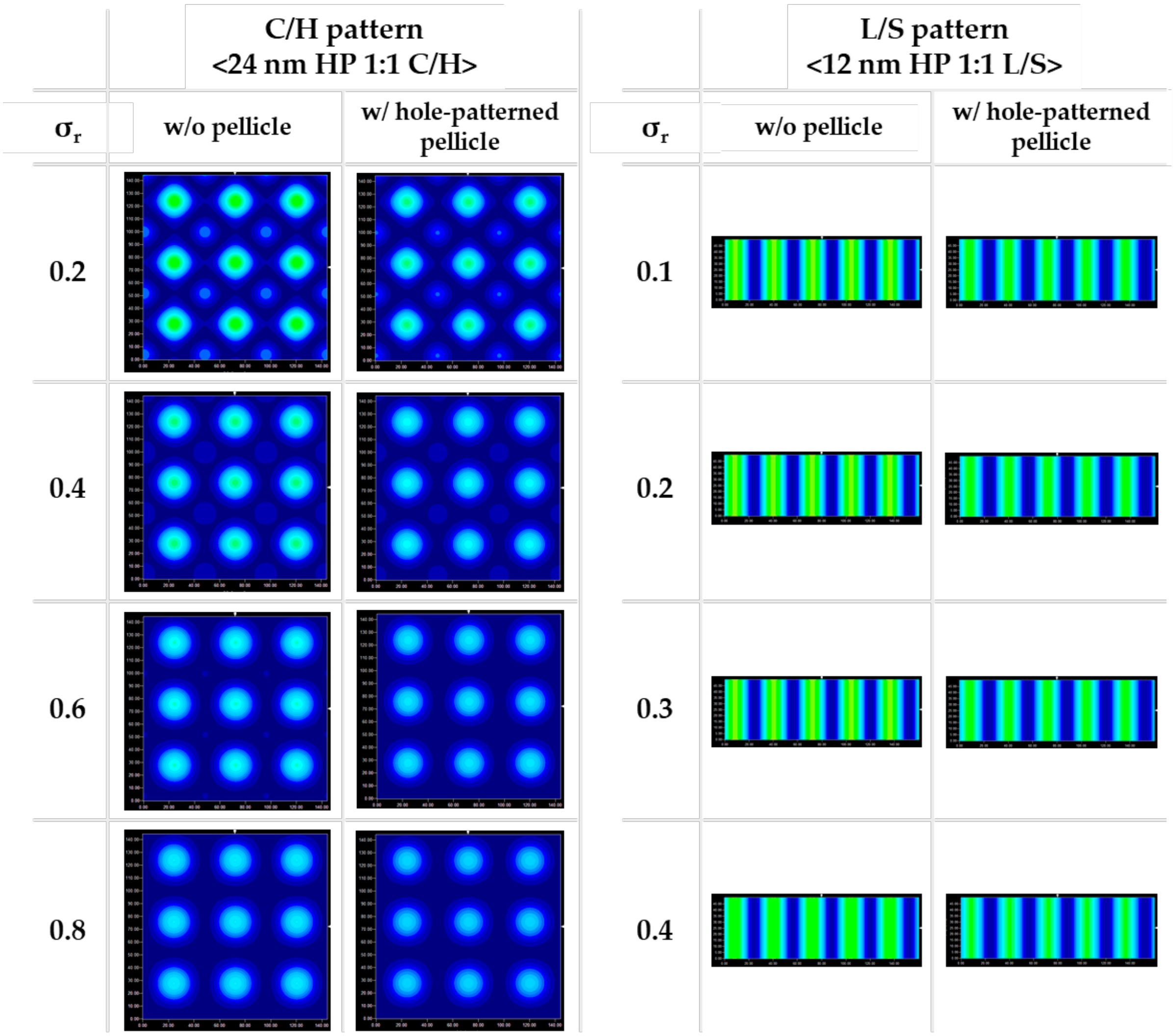

Figure S2. Simulated aerial images for representative scanner-relevant pupil radius sigma values (σ_r = 0.2, 0.4, 0.6, and 0.8 for contact/hole (C/H), σ_r = 0.1, 0.2, 0.3, and 0.4 for line-and-space (L/S)), comparing cases with and without the hole-patterned pellicle for (a) 24 nm half-pitch C/H and (b) 12 nm half-pitch line-and-space (L/S) patterns. The different σ_r ranges reflect the distinct dipole illumination conditions used for the two pattern types, arising from differences in the selected dipole center-sigma (σ_c = 0.6) settings. While the introduction of hole-patterned pellicle results in a reduced overall aerial-image intensity due to finite transmittance, no distinct distortion associated with coherent-interference effects—such as those observed under near-zero sigma conditions in Figure 5—was detected across the investigated σ_r range.
